# 

**Published:** 1919-03-22

**Authors:** 


### Pensions for Hospital Officers and Staffs.

The Report of a Sub-Committee of the Executive Committee of King Edward's Hospital Fund for London has been published by Messrs. C. & E. Layton, 56 Farringdon Street, E.C. 4, and Mr. Geo. Barber, 23 Furnival Street, Holborn, E.C. 4, price 7s. 6d. net. The Report is an important document of 273 foolscap pages. We hope to deal with it fully in the columns of The Hospital at an early date.

### Diet and the Prevention of Influenza.

The Bread and Food Reform League have just published a Memorial signed by eminent members of the medical profession and scientific societies, members of Parliament, clergy, and representative women, asking the Local Government Board, municipal and educational authorities, to direct attention to the following facts. Recent scientific researches show that dietaries lacking in vitamines (essential for the nourishment of the nervous system) found principally in the germ and aleurone cells of cereals, and the anti-scorbutic vitamines, found abundantly in fruits and vegetables, produce deficiency diseased conditions which make people, although apparently in excellent health, physically unfit, and less able to resist epidemic infection, and the Food (War) Committee state that wrong methods of cooking impair the value of anti-scorbutic vitamines. Vegetables should be placed in boiling water, and cooked rapidly for as short a time as possible. *Soda should on no account be added* to the water in which vegetables are cooked or boiled. A part of the "good food" advised to be taken should consist of finely-ground whole wheat meal, or straight run household flour, deprived of bran, but retaining the germ and aleurone cells, oatmeal or other whole cereals. It is advisable that ripe uncooked fruit and well-washed vegetable salads should also be taken daily so as to ensure a supply of the essential vitamines.

Further particulars can be obtained from Miss May Yates, Hon. Sec., Bread and Food Reform League, 37 Essex Street, London, W.C., and funds are urgently needed for this important work.

### HOSPITAL AND INSTITUTIONAL RESULTS FOR 1918.

**Ockenden Convalescent Home, Torquay.**—The substantial legacy of £3,000 left to the home by Mrs. E. Erle, of Tunbridge Wells, makes a considerable impression upon a modest budget.

**The Hospital and Home for Sick Children, Sydenham.**— This institution having extended its area of usefulness by receiving children from all parts of London, has decided to adopt a more distinctive name; in future it will be known as the South-Eastern Hospital for Children. It is hoped that the Whittuck Home at Broadstairs will be reopened soon after the declaration of Peace.

**The West Kent General Hospital, Maidstone.**—For those who have fallen in the war a scheme for the endowment and naming of beds "In Memoriam" has been started. The ordinary expenditure has largely increased, but last year closed with a balance on the right side.

